# Adhesion of *Dictyostelium* Amoebae to Surfaces: A Brief History of Attachments

**DOI:** 10.3389/fcell.2022.910736

**Published:** 2022-05-27

**Authors:** Lucija Mijanović, Igor Weber

**Affiliations:** Division of Molecular Biology, Ruđer Bošković Institute, Zagreb, Croatia

**Keywords:** cell-substratum adhesion, adhesion receptors, cell migration, actin cytoskeleton, amoebozoa, integrins, mesenchymal-amoeboid transition

## Abstract

*Dictyostelium* amoebae adhere to extracellular material using similar mechanisms to metazoan cells. Notably, the cellular anchorage loci in Amoebozoa and Metazoa are both arranged in the form of discrete spots and incorporate a similar repertoire of intracellular proteins assembled into multicomponent complexes located on the inner side of the plasma membrane. Surprisingly, however, *Dictyostelium* lacks integrins, the canonical transmembrane heterodimeric receptors that dominantly mediate adhesion of cells to the extracellular matrix in multicellular animals. In this review article, we summarize the current knowledge about the cell-substratum adhesion in *Dictyostelium*, present an inventory of the involved proteins, and draw parallels with the situation in animal cells. The emerging picture indicates that, while retaining the basic molecular architecture common to their animal relatives, the adhesion complexes in free-living amoeboid cells have evolved to enable less specific interactions with diverse materials encountered in their natural habitat in the deciduous forest soil. Dissection of molecular mechanisms that underlay short lifetime of the cell-substratum attachments and high turnover rate of the adhesion complexes in *Dictyostelium* should provide insight into a similarly modified adhesion phenotype that accompanies the mesenchymal-amoeboid transition in tumor metastasis.

## Introduction

The mid-1980s, when integrins were being unraveled as the receptors that mediate cellular adhesion to extracellular matrix in mammals and other animals (Hynes, 1987, 2004), also witnessed prominent activity in the characterization of the adhesion of unicellular amoeba *Dictyostelium discoideum* to external surfaces (Gingell and Vince, 1982; Owens et al., 1987, 1988). *Dictyostelium* as model organism was on the forefront of the homotypic cell-cell adhesion research (Beug et al., 1973), but its use as a model for the cell-substratum adhesion was hampered by the lack of a well-defined extracellular matrix. The early work was therefore focused on the influence of the physico-chemical characteristics of various glass coatings on cell adhesion. Briefly, it turned out that *Dictyostelium* cells adhered strongly to hydrophobic and positively charged surfaces and weaker to hydrophilic surfaces (Gingell and Vince, 1982; Owens et al., 1988). Of special importance was the introduction of antiadhesive coatings based on entropic repulsion of long polyethylene glycol chains, a concept that has been frequently used ever since to abrogate adhesion of cells to underlying surfaces (Owens et al., 1987; Amiji and Park, 1992). This early body of work by Gingell and co-workers is also significant because of the extensive use of reflection interference contrast microscopy (RICM) and the introduction of internal reflection aqueous fluorescence microscopy for quantitative assessment of adhesion zones (Gingell and Vince, 1982; Todd et al., 1988).

In the 1990s, it was discovered that aggregation-competent cells can migrate with surprisingly little interaction with the substratum, forming discrete attachment spots with a footprint of less than 10% of the cell surface (Wessels et al., 1994; Weber et al., 1995). These attachment zones visible in RICM must, however, be distinguished from much smaller punctuate structures at the bottom cell membrane such as the actin-rich podosomes (Fukui and Inoué, 1997) and the ventral adhesion foci (Patel et al., 2008) (Table 1). A major step forward in the molecular characterization of the adhesion apparatus arrived with the cloning of a *Dictyostelium* talin homologue, talin A (Kreitmeier et al., 1995), which proved to be vital for cell-substratum and EDTA-sensitive cell-cell adhesion (Niewöhner et al., 1997). It was soon discovered that talin A and other proteins such as myosin VII and paxillin B localized to the ventral adhesion foci, the transient punctuate structures at the cell membrane closely apposed to the underlying surface (Kreitmeier et al., 1995; Bukharova et al., 2005; Patel et al., 2008). Their composition, dot-like appearance and immobility relative to the substratum resembled the focal adhesions well-known from fibroblasts and other mammalian cells in culture, which are, however, invariably coupled to transmembrane heterodimeric receptors integrins (Legerstee and Houtsmuller, 2021). Although putative adhesion receptors in *Dictyostelium* were subsequently identified, only SibA showed a limited relatedness to integrin beta subunit, primarily at its C-terminus (Cornillon et al., 2006).

**TABLE 1:** Reported observations and composition of punctuate structures at the cell-substratum interface in *Dictyostelium* cells in chronological order. Abbreviations: IF—immunofluorescence; DIC—differential interference contrast; TEM—transmission electron microscopy; GFP—green fluorescent protein; TIRF—total internal reflection of fluorescence; RICM—reflection interference contrast microscopy; TalA—talin A; TalB—talin B; MyoIB—myosin IB; ABP120—actin binding protein; Arp3—actin-related protein three; PaxB—paxillin B; RasGEF—Ras guanine nucleotide-exchange factor; RapGAP1—Rap GTPase-activating protein one; VinA—vinculin A; CtxI—cortexillin I.

Observation	Protein localization	References
First report on dot-like structures in *Dictyostelium*, ultrastructural evidence for outgrowing actin filaments (actin dots/eupodia)	F-actin (rhodamine-phalloidin)	Yumura and Kitanishi-Yumura, (1990)
Punctuate attachments to the substratum are localized at the filopod tips and underneath cell bodies (ventral foci)	TalA (IF)	Kreitmeier et al. (1995)
Eupodia imaged by DIC microscopy and IF, TalA is not enriched in F-actin-enriched dots (eupodia)	F-actin (IF, rhodamine-phalloidin), TalA (IF), α-actinin (IF), MyoB (IF); the latter two proteins localize to eupodia	Fukui and Inoué, (1997)
Localization of actin-binding proteins and ultrastructure of eupodia (TEM)	F-actin (rhodamine-phalloidin), coronin (IF), fimbrin (IF) and ABP120 (IF) localize to eupodia	Fukui et al. (1999)
Actin dots are sites of close contact to the substratum (RICM) and the anchorage points of the traction force transmission	GFP-actin, average lifetime is 20 s	Uchida and Yumura, (2004)
Actin dots have an average lifetime of 15–20 s	Actin (GFP) and Arp3 (GFP), TIRF	Bretschneider et al. (2004)
Ventral foci are stationary relative to the substratum during cell migration	TalA (GFP) and F-actin (rhodamine-phalloidin) only partially co-localize in the ventral foci	Hibi et al. (2004)
Speckled patterns of staining in regions near the cell membrane closely apposed to the substratum	TalB (IF)	Tsujioka et al. (2004)
Unusually strong accumulation of actin in the dots in *phg2* ^*-*^ cells	Phg2-GFP is not localized to the actin-rich puncta	Gebbie et al. (2004)
PaxB localizes to long lived stationary foci at the cell/substratum interface	PaxB (GFP) is localized to ventral foci distinct from the actin-rich contact dots (ABD-mRFPmars)	Bukharova et al. (2005)
Number of actin dots is increased in RasGEF GbpD-overexpressing cells compared to *GbpD* ^*-*^ cells	F-actin (LimEΔcoil-GFP)	Bosgraaf et al. (2005)
GFP-RapGAP1 localizes to dots stained with TRITC-phalloidin	RapGAP1 (GFP)	Jeon et al. (2007a)
At least two populations of small stationary spots located at the interface of the cells with the substratum	TalA and PaxB are sequentially recruited to discrete ventral foci visible in TIRF	Patel et al. (2008)
Abberant localization and turnover of ventral foci in *frmA* ^*-*^ cells	PaxB (GFP), TalA (GFP)	Patel et al. (2008)
PaxB-containing ventral foci do not form in *talA* ^*-*^ */talB* ^*-*^ cells, but actin-containing dots do form	PaxB (GFP) and actin (GFP)	Tsujioka et al. (2008)
Dots enriched in F-actin are the anchorage points of traction forces	F-actin (GFP-ABD120k)	Iwadate and Yumura, (2008)
VinA and PaxB localize to the ventral foci	VinA (GFP), PaxB (GFP)	Nagasaki et al. (2009)
Spot-like localization of SadA observed by TIRF	SadA (GFP)	Kamprad et al. (2018)

A hallmark of the metastatic spread of tumor cells, the mesenchymal-amoeboid transition, is accompanied by a profound loss of cell adhesion to the extracellular matrix and the disappearance of canonical focal adhesions (Liu et al., 2015). Amoeboid mode of migration in *Dictyostelium* amoeba presents a good model for the migration of metastatic cells in humans, especially since the two organisms share the basic biophysical and regulatory mechanisms responsible for the actin-driven locomotion (Artemenko et al., 2014; Filić et al., 2021). It may seem surprising that much more is known about the structure, assembly, regulation and function of the focal adhesion complexes in mammals than about their apparently much simpler counterparts in *Dictyostelium*. However, the *Dictyostelium* punctuate adhesion contacts are small, fragile and short-lived, and as such still elude more comprehensive characterization. In the following, we provide an inventory of proteins involved in the cell-substratum adhesion in *Dictyostelium* (Table 2).

**TABLE 2:** Knock-out phenotypes and relevant interactors of core proteins involved in the regulation of the cell-substratum adhesion in *Dictyostelium*. The list does not include a number of proteins that were found to influence the adhesion but were not characterized in detail. The phenotype of knock-out strains was described only in the context of the cell-substratum adhesion. The interactions were mostly identified using pull-down and co-immunoprecipitation assays. Additionaly, some binding partners were detected by GDI (guanine nucleotide dissociation inhibitor) and GAP (GTPase-activating protein) assays, RapA activation assay and yeast-two-hybrid assay. Abbreviations: HL5—HL5 nutrient medium; PB—phosphate buffer; MFA—microfluidic assay; CtxI—cortexillin I; TalA—talin A; TalB—talin B; MyoVII—myosin VII; PldB—phospholipase D. An asterisk (*) indicates conflicting reports.

Protein	Knock-out Phenotype	Interactors	References
Phg1A	reduced attachment to glass in HL5; SibA mRNA and total protein levels low, SibA surface levels low		Cornillon et al. (2000), Benghezal et al. (2003), Gebbie et al. (2004), Froquet et al. (2012)
Phg1B	reduced attachment to glass in HL5 (thermosensitive defect); double *phg1A* ^*-*^/*phg1B* ^*−*^—reduced attachment to glass in HL5, slightly reduced in PB		Benghezal et al. (2003)
SadA	abolished attachment to plastic in HL5; reduced attachment to glass in PB (MFA); SibA protein surface levels low, total level of SibA low	CtxI	Fey et al. (2002), Kowal and Chisholm (2011), Froquet et al. (2012), Tarantola et al. (2014)
SibA	reduced attachment to glass in PB (MFA)	TalA	Cornillon et al. (2006), Tarantola et al. (2014)
Phg2	reduced attachment to plastic in HL5; reduced attachment to glass in HL5; increased attachment in PB on filters	RapA	Gebbie et al. (2004), Kortholt et al. (2006), Jeon et al. (2007b)
Talin A	reduced attachment to plastic in HL5*; normal attachment to plastic in HL5*; reduced attachment to glass in HL5; reduced attachment to glass in PB (also in MFA); reduced attachment to albumin-coated glass	MyoVII; Sib family proteins (SibA-SibE)	Niewöhner et al. (1997)*; Simson et al. (1998), Gebbie et al. (2004), Tuxworth et al. (2005), Cornillon et al. (2006), Ibarra et al. (2006), Kortholt et al. (2006)*, Tarantola et al. (2014), Plak et al. (2016)*
Talin B	normal attachment to plastic in HL5; double *talA* ^*-*^ */talB* ^*-*^—abolished attachment to substratum in HL5	RapA	Patel et al. (2008), Plak et al. (2016)
Paxillin B	reduced attachment to plastic in HL5; reduced attachment to glass in PB; expression of PldB restores wild-type adhesion levels	PldB	Bukharova et al. (2005), Patel et al. (2008), Nagasaki et al. (2009), Pribic et al. (2011)
Myosin VII	reduced attachment to plastic in HL5; strongly reduced attachment to glass in HL5; TalA levels reduced	TalA	Titus (1999), Tuxworth et al. (2001), Tuxworth et al. (2005), Gebbie et al. (2004), Ibarra et al. (2006), Kortholt et al. (2006), Galdeen et al. (2007)
FrmA	increased attachment to plastic in HL5; mislocalization and altered turnover of TalA and PaxB in adhesion foci		Patel et al. (2008)
RapA	lethal; *knock-down via antisense RNA—decreased growth and viability*	PI3K; Phg2; GbpD; RapGAP1; RapGAPB; TalB	Kang et al. (2002), Gebbie et al. (2004), Kortholt et al. (2006), Kortholt et al. (2010), Jeon et al. (2007a), Jeon et al. (2007b), Parkinson et al. (2009), Plak et al. (2016)
GbpD	reduced attachment to plastic in PB	RapA	Bosgraaf et al. (2005), Kortholt et al. (2006)
RapGAP1	increased attachment in PB on filters	RapA	Jeon et al. (2007a)
RapGAPB	increased attachment to plastic in HL5	RapA	Parkinson et al. (2009)
RapC	increased attachment in PB on filters		Park et al. (2018a), Jeon et al. (2021), Kim et al. (2021)

## Molecular Inventory: Adhesion Receptors and Their Binding Partners

After the identification of a crucial role played by a *Dictyostelium* talin orthologue in cell-substratum adhesion (Niewöhner et al., 1997), it appeared plausible that this process relied on a variant of integrin-mediated adhesive complexes (IACs), similar to those found in Metazoa (Kang et al., 2021). A quest to identify candidate adhesion receptors began by a screen of randomly generated mutants for defects in phagocytosis (Cornillon et al., 2000). The screen exposed Phg1A, a protein belonging to the transmembrane nine family (TM9) with the typical large extracellular domain and nine hydrophobic C-terminal transmembrane domains (Froquet et al., 2008), soon followed by the discovery of related TM9 proteins Phg1B and Phg1C (Benghezal et al., 2003). Characterization of single and double knock-outs of Phg1A and Phg1B revealed a severe defect in adhesion of *phg1A* ^*-*^ cells to hydrophilic particles and suggested a synergistic effects of the two proteins in controlling the adhesion (Cornillon et al., 2000; Benghezal et al., 2003). Concomitantly to TM9 proteins, yet another protein with nine putative transmembrane domains, SadA, was shown to be important for adhesion in *Dictyostelium*. *sadA* ^*-*^ cells were unable to attach to plastic dishes and to spread, and their phagocytosis was strongly impaired (Fey et al., 2002; Froquet et al., 2012). Since SadA contains three conserved extracellular EGF-like repeats that are also present in integrins and tenascins, it was suggested that it represents a genuine adhesion receptor (Fey et al., 2002). It was later shown that the cytoplasmic tail of SadA interacts with the talin A/myosin VII complex (Tuxworth et al., 2005; Cornillon et al., 2006), and with cortexillin I (Kowal and Chisholm, 2011), indicating a link to the actin cytoskeleton.

The most likely candidate for the main adhesion receptor in *Dictyostelium*, however, was identified in another random mutagenesis screen and named SibA (similar to integrin beta A) (Cornillon et al., 2006). As indicated by its name, SibA is a type I transmembrane protein with features similar to metazoan integrin β-chains, e.g. an extracellular Von Willebrandt A domain and a single glycine-rich transmembrane domain, but also with significant structural differences in comparison to integrin β (Cornillon et al., 2006). There are four close homologs of SibA (SibB to SibE) in *Dictyostelium* and all of them bind talin A via their cytosolic domain (Cornillon et al., 2006). Only SibA and SibC are expressed abundantly in vegetative cells and their individual genetic inactivation caused a partial loss of adhesion to various substrata and particles, but generation of a double *sibA* ^*-*^/*sibC* ^*-*^ knockout strain failed, suggesting possible lethality (Cornillon et al., 2008). Taken together, the present state of knowledge about the transmembrane proteins involved in the regulation of adhesion to external surfaces suggests that Sib proteins are the primary receptors, whereas SadA and Phg1 proteins play an auxiliary and regulatory role. The expression level, stability and targeting of SibA to the cell surface are influenced by Phg1A and SadA (Froquet et al., 2012). Compared to wild-type, SibA, SadA and Phg1 deficient cells exhibit comparable defects in phagocytosis and the adhesion to underlying surfaces (Fey et al., 2002; Benghezal et al., 2003; Froquet et al., 2012; Tarantola et al., 2014).

The phosphorylation of functional motifs in the cytoplasmic integrin tails leads to conformational changes that enable the recruitment of downstream proteins such as 14-3-3, talin, and kindlin (Gahmberg and Grönholm, 2022). Most integrin β-tails contain conserved NPXY motifs that belong to a recognition sequence for phosphotyrosine-binding (PTB) domains (Calderwood et al., 2003). Besides PTBs, Src homology 2 (SH2) domains also bind phosphorylated tyrosine and are important in integrin signaling (Gahmberg and Grönholm, 2022). Instead by canonical tyrosine kinases, tyrosine phosphorylation in *Dictyostelium* is mediated by tyrosine kinase-like (TLK) proteins (Goldberg et al., 2006). While some TLKs are known to phosphorylate tyrosines exclusively, e.g. ZakA and Dpyk2-4, the others are dual-specificity kinases, e.g. SplA and Shk1 (Goldberg et al., 2006). The prime adhesion receptor in *Dictyostelium*, SibA, contains two NPXY motifs (Cornillon et al., 2006). It was shown that the membrane-proximal NPXY motif is essential for binding of SibA to talin A similar to situation in metazoa, thus suggesting that SibA might be regulated in a manner analogous to integrins (Cornillon et al., 2006; Anthis et al., 2009).

FERM (the four-point-one, ezrin, radixin and moesin) domain proteins encompass, together with paxillin and vinculin, the core members of IAC in metazoans (Calderwood et al., 2013), and seven have been identified in *Dictyostelium*: talin A and B, myosin VII, myosin G, and FrmA-C (Patel et al., 2008; Breshears et al., 2010). The importance for the cell-substratum adhesion of the first characterized member, talin A, lived up to expectations: *talA* ^*-*^ cells showed varying degrees of weakened adhesion to multiple substrata (Niewöhner et al., 1997; Simson et al., 1998; Gebbie et al., 2004; Tarantola et al., 2014), and defective uptake of various particles (Niewöhner et al., 1997; Gebbie et al., 2004). Talin A is the first protein to show up in the nascent ventral adhesion foci and at the distal ends of attached filopodia (Kreitmeier et al., 1995; Patel et al., 2008), but is also present at the trailing regions of locomoting cells (Hibi et al., 2004; Tuxworth et al., 2005; Tsujioka et al., 2012). Cells lacking another talin paralog in *Dictyostelium*, talin B, show only slightly impaired adhesion to the substratum, but when both talins are inactivated, the double KO cells are unable to attach at all when cultivated in the HL5 nutrient medium (Tsujioka et al., 2008; Plak et al., 2016). Paxillin B is a close homolog of mammalian paxillin, contains four highly conserved LIM domains and four paxillin LD domains, and is recruited to the tips of filopodia and the ventral adhesion foci sequentially after talin A (Bukharova et al., 2005; Patel et al., 2008; Nagasaki et al., 2009). Cells lacking paxillin B are defective in adhesion to substrata, but, interestingly, its overexpression was also reported to impair adhesion (Bukharova et al., 2005; Duran et al., 2009; Nagasaki et al., 2009). A class VII myosin is also enriched in the ventral adhesion foci and the filopodia tips (Tuxworth et al., 2001; Petersen et al., 2016), whereas the *myoVII* ^*-*^ cells show reduced attachment areas and diminished binding to particles (Tuxworth et al., 2001; Gebbie et al., 2004). *Dictyostelium* vinculins A and B possess regions with binding sites for α-actinin, talin, paxillin and actin, similar to human vinculin (Nagasaki et al., 2009; Huber and O’Day, 2012). Vinculin A appears to localize to the ventral adhesion foci, but its importance for the cell-substratum adhesion in general has not been investigated, apart from its requirement for cytokinesis in cells devoid of a functional myosin II (Nagasaki et al., 2009).

Similar localizations and mutant phenotypes indicated that talin A, paxillin B and myosin VII belong to the same complex, probably related to metazoan IACs. Indeed, it was shown that talin A is stabilized against degradation via its interaction with the myosin VII tail (Gebbie et al., 2004; Tuxworth et al., 2005; Galdeen et al., 2007). Although the two proteins do not depend on each other for localization (Tuxworth et al., 2005), formation of the complex prolongs the residence of myosin VII on the plasma membrane (Galdeen et al., 2007). FrmA is required for the proper cell-substratum adhesion by promoting the turnover of the ventral adhesion structures (Patel et al., 2008). In *frmA* ^−^ cells, the ventral adhesion foci containing paxillin B localize aberrantly around the circumference of the cell-substratum contact area and the persistence of these foci increases greatly, which is probably responsible for an increased adhesion of the mutant cells to the substratum (Patel et al., 2008). On the other hand, *frmB* ^−^ cells have a significantly reduced adhesion (Kim et al., 2017). Multiple evidence about functional analogies and interactions between the FERM and the transmembrane proteins strengthens the notion that they assemble into transient structures of the IAC type. For instance, the talin A/myosin VII complex interacts with the conserved cytosolic domain of Sib family proteins (Tuxworth et al., 2005; Cornillon et al., 2006). Also, adhesion defects of *talA* ^*-*^ and *myoVII* ^*-*^ cells are more pronounced on hydrophilic substrata, similar to those of *phg1* ^*-*^ and *phg2* ^*-*^ cells, suggesting an involvement in the same process (Gebbie et al., 2004). Small GTPase Rap1 is considered to be one of the key regulators of cell adhesion in Metazoa via its direct and indirect interactions with talin (Calderwood et al., 2013; Zhang et al., 2014; Zhu et al., 2017). In *Dictyostelium* too, RapA interacts directly with the RA domain of talin B, and regulates talin B signaling by local allosteric activation rather than by its recruitment (Plak et al., 2016). Since the deletion of RapA is likely lethal (Kang et al., 2002; Jeon et al., 2007b), data about the role of RapA in cell adhesion are based on overexpressor strains and genetic deletion of its regulators. Overexpression of active RapA in wild-type cells strongly increases adhesion (Rebstein et al., 1993; Jeon et al., 2007b, 2021), in single *talA* ^*-*^ or *talB* ^*-*^ the effect is modest, while in double mutants there is no effect, suggesting that RapA regulates cell-substratum adhesion also via an indirect activation of Talin A (Plak et al., 2016), similar to the activation of Talin1 by RIAM proteins in mammalian cells (Lagarrigue et al., 2016). Consistent with these results, cells lacking the RapGEF GbpD are more loosely attached to the substratum compared to wild-type cells, while activation of RapA by GbpD leads to a stronger attachment (Bosgraaf et al., 2005; Kortholt et al., 2006). Further corroboration of a positive role played by RapA in promoting cell-substratum adhesion comes from the studies of RapGAP proteins. So, the lack of RapGAP1, as well as of RapGAPB, leads to an increase in cell attachment, whereas RapGAP1-overexpressing cells are weakly attached to the substratum (Jeon et al., 2007a; Parkinson et al., 2009).

## Other Liaisons

Attachment of cells to external surfaces, especially during locomotion, depends also on other processes within the actin cytoskeleton in addition to adhesion. A classic example is the myosin II-driven contractility that supports detachment of the rear end of migrating cells (Jay et al., 1995). It has thus been proposed that RapA, in addition to its talin-mediated role, negatively controls myosin II assembly through the activation of the serine/threonine kinase Phg2 specifically at the leading edges of migrating and dividing cells (Kortholt et al., 2006; Jeon et al., 2007a). Indeed, *phg2* ^*-*^ cells are strongly impaired in cell-substratum adhesion and form exceedingly large assemblies of F-actin at the ventral cell surface, suggesting an additional actin depolymerization activity of Phg2 (Gebbie et al., 2004), possibly related to the established role of mammalian serine/threonine kinases in the regulation of integrin-mediated adhesion (Bachmann et al., 2019). Another evidence that the spatial balance in actin polymerization influences cell adhesion comes from the cells lacking a functional SCAR/WAVE complex, which show reduced cell–substratum interactions in migration, although they still possess actin pseudopods (Veltman et al., 2012). This finding thus indicates a specific role of SCAR/WAVE in regulating the strength of the cellular traction stresses (Bastounis et al., 2011). Cells lacking a component of the SCAR/WAVE complex NapA have an even more pronounced adhesion defect, suggesting additional involvement of NapA in a SCAR/WAVE-independent pathway (Ibarra et al., 2006).

A number of other proteins were connected to the regulation of the cell-substratum adhesion in *Dictyostelium*, but the mechanisms of their action have not been clarified. For example, RapC is a close homolog of RapA with antagonistic functions in cell adhesion and migration, since *rapC* ^*-*^ cells show an increased substratum adhesion (Park et al., 2018a; Kim et al., 2021). Interestingly, mammalian Rap2 was found to promote integrin-dependent adhesion similar to Rap1 (McLeod et al., 2004). Increased cell-substratum adhesion has also been detected in *Dictyostelium* cells lacking AmpA and Sma (Kelsey and Blumberg, 2013), SepA (Müller-Taubenberger et al., 2009), coronin 7 (Shina et al., 2010b), dynamin B (Rai et al., 2011), copine A (Buccilli et al., 2019), KrsB (Artemenko et al., 2012), SpdA (Dias et al., 2016), LrrkA (Bodinier et al., 2021), and in mutants identified in a screen for increased cell attachment, e.g. PTEN, HtmA, AraA, DspA, and AbnC (Lampert et al., 2017). In mammals, coronin 1 is important for integrin β2 translocation to the platelet surface (Riley et al., 2020), dynamin 2 was shown to control Rap1 activation via FAK/Pyk2 and RapGEF leading to integrin clustering in T lymphocytes (Eppler et al., 2017), whereas PTEN reduces tyrosine phosphorylation by FAK and thereby negatively regulates the formation of focal adhesions and spreading in fibroblasts (Tamura et al., 1998). Conversely, diminished adhesion of *Dictyostelium* cells has been reported after the knock-out of genes coding for Gp130 (Chia et al., 2005), Ate1 (Batsios et al., 2019), Cbp7 (Park et al., 2018b), ForH (Schirenbeck et al., 2005), Ino1 (Frej et al., 2016), LrrA (Liu et al., 2005), and SecG (Shina et al., 2010a). Cytohesin 1, a mammalian homolog of SecG, was shown to bind β2 integrin chain in T lymphocytes (Kolanus et al., 1996). Mutant *Dictyostelium* cells devoid of IBARa have been described as lacking the dynamic spreading behavior characteristic for wild-type cells (Linkner et al., 2014).

## Concluding Remarks

Among over 200 human adhesome proteins, fewer than 50 are found in *Dictyostelium* (Zaidel-Bar, 2009). Although the adhesive properties of *Dictyostelium* cells are remarkably similar to those of animals, most *Dictyostelium* adhesion molecules have little sequence similarity to animal proteins (Abedin and King, 2010). However, over the past decade, integrins and other components of the IAC (or adhesome) were identified outside of Metazoa, leading to the suggestion that the IAC and associated proteins have a more ancient evolutionary origin than previously anticipated (Abedin and King, 2010; Sebé-Pedrós et al., 2010; Kang et al., 2021), similar to proteins involved in other cell adhesion systems (Harwood and Coates, 2004; Murray and Zaidel-Bar, 2014). Based on the sequenced genomes of *Dictyostelium discoideum* and *Acanthamoeba castellanii*, it was thought until recently that integrins α and β were not represented in Amoebozoa (Cavalier-Smith, 2017). However, a recent examination of 113 genomes and transcriptomes identified integrin α homologs in 23 and integrin β homologs in 19 amoebozoan taxa (Kang et al., 2021). Peculiarly, no evidence of integrins was found in the few lineages of Amoebozoa that aggregate to form tissue-like assemblies, such as Dictyostelida, although they produce an elaborate ECM during multicellular development (Huber and O’Day, 2017). It is probable that this is due to a secondary loss of integrins and other IAC components in majority of amoebozoan taxa, similar to the situation in the closest relative of animals, Choanoflagellata, which also lack integrins. One should also not completely dismiss a tantalizing possibility that some IAC components, including integrins, were lost during axenic selection of common laboratory strains such as the AX4 whose genome was sequenced (Eichinger et al., 2005; Bloomfield et al., 2008). Since integrin repeats were identified in two phyla of Asgard archaea (Liu et al., 2021), it is highly likely that integrins were present in the common ancestor of Amoebozoa and Metazoa. Very little is known about the function of amoebozoan integrins, but they are probably involved in the adhesion to external surfaces as in unicellular holozoans (Custodio et al., 1995; Parra-Acero et al., 2020). Interestingly, many amoebozoan species contain either integrins α or β, and yet possess all the signaling components of the IAC (Kang et al., 2021). It would therefore be interesting to examine the functional consequences of a possible integrin homodimerization in these organisms.

It would be of immediate interest to invest more work into the characterization of *Dictyostelium* adhesome/IAC. One obvious strategy would be to use the powerful tools of proteomics that were utilized to establish a consensus core adhesome of 60 proteins in mammals (Horton et al., 2015). A complementary, ultrastructural approach should be used to obtain a three-dimensional reconstruction of ventral focal adhesion complexes using cryo-electron tomography (Patla et al., 2010). It has been proposed that the focal adhesions mediate the cell attachment by suppressing the repulsive thermal undulations of the cell plasma membrane (Zidovska and Sackmann, 2006; Huang et al., 2012; Fenz et al., 2017). The use of discrete, punctuate adhesive contacts between the cell and its substratum appears to be a universal strategy to accomplish a contact between the two surfaces with a minimal investment of multiprotein assemblies. The emerging *Dictyostelium* adhesome and a relative ease in manipulating the adhesive conditions in this organism might provide a fruitful independent testing ground of this concept (Loomis et al., 2012).

## References

[B1] Abedin M. King N. (2010). Diverse Evolutionary Paths to Cell Adhesion. Trends Cell Biol. 20, 734–742. 10.1016/j.tcb.2010.08.002 20817460 PMC2991404

[B2] Amiji M. Park K. (1992). Prevention of Protein Adsorption and Platelet Adhesion on Surfaces by PEO/PPO/PEO Triblock Copolymers. Biomaterials 13, 682–692. 10.1016/0142-9612(92)90128-B 1420713

[B3] Anthis N. J. Haling J. R. Oxley C. L. Memo M. Wegener K. L. Lim C. J. (2009). β Integrin Tyrosine Phosphorylation Is a Conserved Mechanism for Regulating Talin-Induced Integrin Activation. J. Biol. Chem. 284, 36700–36710. 10.1074/jbc.M109.061275 19843520 PMC2794784

[B4] Artemenko Y. Batsios P. Borleis J. Gagnon Z. Lee J. Rohlfs M. (2012). Tumor Suppressor Hippo/MST1 Kinase Mediates Chemotaxis by Regulating Spreading and Adhesion. Proc. Natl. Acad. Sci. U.S.A. 109, 13632–13637. 10.1073/pnas.1211304109 22847424 PMC3427065

[B5] Artemenko Y. Lampert T. J. Devreotes P. N. (2014). Moving towards a Paradigm: Common Mechanisms of Chemotactic Signaling in Dictyostelium and Mammalian Leukocytes. Cell Mol. Life Sci. 71, 3711–3747. 10.1007/s00018-014-1638-8 24846395 PMC4162842

[B6] Bachmann M. Kukkurainen S. Hytönen V. P. Wehrle-Haller B. (2019). Cell Adhesion by Integrins. Physiol. Rev. 99, 1655–1699. 10.1152/physrev.00036.2018 31313981

[B7] Bastounis E. Meili R. Alonso-Latorre B. del Álamo J. C. Lasheras J. C. Firtel R. A. (2011). The SCAR/WAVE Complex Is Necessary for Proper Regulation of Traction Stresses during Amoeboid Motility. Mol. Biol. Cell 22, 3995–4003. 10.1091/mbc.e11-03-0278 21900496 PMC3204062

[B8] Batsios P. Ishikawa-Ankerhold H. C. Roth H. Schleicher M. Wong C. C. L. Müller-Taubenberger A. (2019). Ate1-mediated Posttranslational Arginylation Affects Substrate Adhesion and Cell Migration in Dictyostelium discoideum. Mol. Biol. Cell 30, 453–466. 10.1091/mbc.E18-02-0132 30586322 PMC6594445

[B9] Benghezal M. Cornillon S. Gebbie L. Alibaud L. Brückert F. Letourneur F. (2003). Synergistic Control of Cellular Adhesion by Transmembrane 9 Proteins. Mol. Biol. Cell 14, 2890–2899. 10.1091/mbc.e02-11-0724 12857872 PMC165684

[B10] Beug H. Katz F. E. Gerisch G. (1973). Dynamics of Antigenic Membrane Sites Relating to Cell Aggregation in Dictyostelium discoideum. J. Cell Biol. 56, 647–658. 10.1083/JCB.56.3.647 4631665 PMC2108928

[B11] Bloomfield G. Tanaka Y. Skelton J. Ivens A. Kay R. R. (2008). Widespread Duplications in the Genomes of Laboratory Stocks of Dictyostelium discoideum. Genome Biol. 9, R75. 10.1186/gb-2008-9-4-r75 18430225 PMC2643946

[B12] Bodinier R. Sabra A. Leiba J. Marchetti A. Lamrabet O. Ayadi I. (2021). Role of LrrkA in the Control of Phagocytosis and Cell Motility in Dictyostelium discoideum. Front. Cell Dev. Biol. 9, 1–11. 10.3389/fcell.2021.629200 PMC7982419 33763419

[B13] Bosgraaf L. Waijer A. Engel R. Visser A. J. W. G. Wessels D. Soll D. (2005). RasGEF-containing Proteins GbpC and GbpD Have Differential Effects on Cell Polarity and Chemotaxis in Dictyostelium. J. Cell Sci. 118, 1899–1910. 10.1242/jcs.02317 15827084

[B14] Breshears L. M. Wessels D. Soll D. R. Titus M. A. (2010). An Unconventional Myosin Required for Cell Polarization and Chemotaxis. Proc. Natl. Acad. Sci. U.S.A. 107, 6918–6923. 10.1073/pnas.0909796107 20351273 PMC2872422

[B15] Bretschneider T. Diez S. Anderson K. Heuser J. Clarke M. Müller-Taubenberger A. (2004). Dynamic Actin Patterns and Arp2/3 Assembly at the Substrate-Attached Surface of Motile Cells. Curr. Biol. 14, 1–10. 10.1016/j.cub.2003.12.005 14711408

[B16] Buccilli M. J. Ilacqua A. N. Han M. Banas A. A. Wight E. M. Mao H. (2019). Copine A Interacts with Actin Filaments and Plays a Role in Chemotaxis and Adhesion. Cells 8, 758. 10.3390/cells8070758 PMC6679068 31330887

[B17] Bukahrova T. Weijer G. Bosgraaf L. Dormann D. van Haastert P. J. Weijer C. J. (2005). Paxillin Is Required for Cell-Substrate Adhesion, Cell Sorting and Slug Migration during Dictyostelium Development. J. Cell Sci. 118, 4295–4310. 10.1242/jcs.02557 16155255

[B18] Calderwood D. A. Campbell I. D. Critchley D. R. (2013). Talins and Kindlins: Partners in Integrin-Mediated Adhesion. Nat. Rev. Mol. Cell Biol. 14, 503–517. 10.1038/nrm3624 23860236 PMC4116690

[B19] Calderwood D. A. Fujioka Y. De Pereda J. M. García-Alvarez B. Nakamoto T. Margolis B. (2003). Integrin β Cytoplasmic Domain Interactions with Phosphotyrosine-Binding Domains: A Structural Prototype for Diversity in Integrin Signaling. Proc. Natl. Acad. Sci. U.S.A. 100, 2272–2277. 10.1073/pnas.262791999 12606711 PMC151330

[B20] Cavalier-Smith T. (2017). Origin of Animal Multicellularity: Precursors, Causes, Consequences-The Choanoflagellate/sponge Transition, Neurogenesis and the Cambrian Explosion. Phil. Trans. R. Soc. B 372, 20150476. 10.1098/rstb.2015.0476 28242741 PMC5332866

[B21] Chia C. P. Gomathinayagam S. Schmaltz R. J. Smoyer L. K. (2005). Glycoprotein Gp130 of Dictyostelium discoideum Influences Macropinocytosis and Adhesion. Mol. Biol. Cell 16, 2681–2693. 10.1091/mbc.e04-06-0483 15788570 PMC1142416

[B22] Cornillon S. Froquet R. Cosson P. (2008). Involvement of Sib Proteins in the Regulation of Cellular Adhesion in Dictyostelium discoideum. Eukaryot. Cell 7, 1600–1605. 10.1128/EC.00155-08 18676957 PMC2547077

[B23] Cornillon S. Gebbie L. Benghezal M. Nair P. Keller S. Wehrle‐Haller B. (2006). An Adhesion Molecule in Free‐Living Dictyostelium Amoebae with Integrin β Features. EMBO Rep. 7, 617–621. 10.1038/sj.embor.7400701 16699495 PMC1479592

[B24] Cornillon S. Pech E. Benghezal M. Ravanel K. Gaynor E. Letourneur F. (2000). Phg1p Is a Nine-Transmembrane Protein Superfamily Member Involved in Dictyostelium Adhesion and Phagocytosis. J. Biol. Chem. 275, 34287–34292. 10.1074/jbc.M006725200 10944536

[B25] Custodio M. R. Imsiecke G. Borojevic R. Rinkevich B. Rogerson A. Müller W. E. G. (1995). Evolution of Cell Adhesion Systems: Evidence for Arg-Gly-Asp-Mediated Adhesion in the Protozoan Neoparamoeba Aestuarina. J. Eukaryot. Microbiol. 42, 721–724. 10.1111/j.1550-7408.1995.tb01623.x 8520588

[B26] Dias M. Brochetta C. Marchetti A. Bodinier R. Brückert F. Cosson P. (2016). Role of SpdA in Cell Spreading and Phagocytosis in Dictyostelium. PLoS One 11, e0160376–17. 10.1371/journal.pone.0160376 27512991 PMC4981364

[B27] Duran M. B. Rahman A. Colten M. Brazill D. (2009). Dictyostelium discoideum Paxillin Regulates Actin-Based Processes. Protist 160, 221–232. 10.1016/j.protis.2008.09.005 19213599 PMC2743336

[B28] Eichinger L. Pachebat J. A. Glöckner G. Rajandream M. A. Sucgang R. Berriman M. (2005). The Genome of the Social Amoeba Dictyostelium discoideum. Nature 435, 43–57. 10.1038/nature03481 15875012 PMC1352341

[B29] Eppler F. J. Quast T. Kolanus W. (2017). Dynamin2 Controls Rap1 Activation and Integrin Clustering in Human T Lymphocyte Adhesion. PLoS One 12, e0172443–30. 10.1371/journal.pone.0172443 28273099 PMC5342215

[B30] Fenz S. F. Bihr T. Schmidt D. Merkel R. Seifert U. Sengupta K. (2017). Membrane Fluctuations Mediate Lateral Interaction between Cadherin Bonds. Nat. Phys. 13, 906–913. 10.1038/nphys4138

[B31] Fey P. Stephens S. Titus M. A. Chisholm R. L. (2002). SadA, a Novel Adhesion Receptor in Dictyostelium. J. Cell Biol. 159, 1109–1119. 10.1083/jcb.200206067 12499361 PMC2173991

[B32] Filić V. Mijanović L. Putar D. Talajić A. Ćetković H. Weber I. (2021). Regulation of the Actin Cytoskeleton via Rho Gtpase Signalling in Dictyostelium and Mammalian Cells: A Parallel Slalom. Cells 10, 1592. 10.3390/cells10071592 34202767 PMC8305917

[B33] Frej A. D. Clark J. Le Roy C. I. Lilla S. Thomason P. A. Otto G. P. (2016). The Inositol-3-Phosphate Synthase Biosynthetic Enzyme Has Distinct Catalytic and Metabolic Roles. Mol. Cell Biol. 36, 1464–1479. 10.1128/mcb.00039-16 26951199 PMC4859692

[B34] Froquet R. Cherix N. Birke R. Benghezal M. Cameroni E. Letourneur F. (2008). Control of Cellular Physiology by TM9 Proteins in Yeast and Dictyostelium. J. Biol. Chem. 283, 6764–6772. 10.1074/jbc.M704484200 18178563

[B35] Froquet R. le Coadic M. Perrin J. Cherix N. Cornillon S. Cosson P. (2012). TM9/Phg1 and SadA Proteins Control Surface Expression and Stability of SibA Adhesion Molecules in Dictyostelium. Mol. Biol. Cell 23, 679–686. 10.1091/mbc.E11-04-0338 22219373 PMC3279395

[B36] Fukui Y. De Hostos E. Yumura S. Kitanishi-Yumura T. Inoué S. (1999). Architectural Dynamics of F-Actin in Eupodia Suggests Their Role in Invasive Locomotion in Dictyostelium. Exp. Cell Res. 249, 33–45. 10.1006/excr.1999.4445 10328951

[B37] Fukui Y. Inoué S. (1997). Amoeboid Movement Anchored by Eupodia, New Actin-Rich Knobby Feet in Dictyostelium. Cell Motil. Cytoskelet. 36, 339–354. 10.1002/(SICI)1097-0169 9096956

[B38] Gahmberg C. G. Grönholm M. (2022). How Integrin Phosphorylations Regulate Cell Adhesion and Signaling. Trends Biochem. Sci. 47, 265–278. 10.1016/j.tibs.2021.11.003 34872819 PMC8642147

[B39] Galdeen S. A. Stephens S. Thomas D. D. Titus M. A. (2007). Talin Influences the Dynamics of the Myosin VII-Membrane Interaction. Mol. Biol. Cell 18, 4074–4084. 10.1091/mbc.e06-07-0586 17671169 PMC1995725

[B40] Gebbie L. Benghezal M. Cornillon S. Froquet R. Cherix N. Malbouyres M. (2004). Phg2, a Kinase Involved in Adhesion and Focal Site Modeling in Dictyostelium. Mol. Biol. Cell 15, 3915–3925. 10.1091/mbc.E03-12-0908 15194808 PMC491846

[B41] Gingell D. Vince S. (1982). Substratum Wettability and Charge Influence the Spreading of Dictyostelium Amoebae and the Formation of Ultrathin Cytoplasmic Lamellae. J. Cell Sci. 54, 255–285. 10.1242/jcs.54.1.255

[B42] Goldberg J. M. Manning G. Liu A. Fey P. Pilcher K. E. Xu Y. (2006). The Dictyostelium Kinome-Analysis of the Protein Kinases from a Simple Model Organism. PLoS Genet. 2, e38. 10.1371/journal.pgen.0020038 16596165 PMC1420674

[B43] Harwood A. Coates J. C. (2004). A Prehistory of Cell Adhesion. Curr. Opin. Cell Biol. 16, 470–476. 10.1016/j.ceb.2004.07.011 15363795

[B44] Hibi M. Nagasaki A. Takahashi M. Yamagishi A. Uyeda T. Q. P. (2004). Dictyostelium discoideum Talin A Is Crucial for Myosin II-independent and Adhesion-dependent Cytokinesis. J. Muscle Res. Cell Motil. 25, 127–140. 10.1023/B:JURE.0000035842.71415.f3 15360128

[B45] Horton E. R. Byron A. Askari J. A. Ng D. H. J. Millon-Frémillon A. Robertson J. (20152015). Definition of a Consensus Integrin Adhesome and its Dynamics during Adhesion Complex Assembly and Disassembly. Nat. Cell Biol. 17, 1577–1587. 10.1038/ncb3257 PMC4663675 26479319

[B46] Huang J. Peng X. Xiong C. Fang J. (2012). Influence of Substrate Rigidity on Primary Nucleation of Cell Adhesion: A Thermal Fluctuation Model. J. Colloid Interface Sci. 366, 200–208. 10.1016/j.jcis.2011.09.046 21999957

[B47] Huber R. J. O'Day D. H. (2012). EGF-like Peptide-Enhanced Cell Movement in Dictyostelium Is Mediated by Protein Kinases and the Activity of Several Cytoskeletal Proteins. Cell Signal. 24, 1770–1780. 10.1016/j.cellsig.2012.05.004 22588127

[B48] Huber R. J. O'Day D. H. (2017). Extracellular Matrix Dynamics and Functions in the Social Amoeba Dictyostelium: A Critical Review. Biochimica Biophysica Acta (BBA) - General Subj. 1861, 2971–2980. 10.1016/j.bbagen.2016.09.026 27693486

[B49] Hynes R. (1987). Integrins: A Family of Cell Surface Receptors. Cell 48, 549–554. 10.1016/0092-8674(87)90233-9 3028640

[B50] Hynes R. O. (2004). The Emergence of Integrins: a Personal and Historical Perspective. Matrix Biol. 23, 333–340. 10.1016/J.MATBIO.2004.08.001 15533754 PMC3493146

[B51] Ibarra N. Blagg S. L. Vazquez F. Insall R. H. (2006). Nap1 Regulates Dictyostelium Cell Motility and Adhesion through SCAR-dependent and -Independent Pathways. Curr. Biol. 16, 717–722. 10.1016/j.cub.2006.02.068 16581519

[B52] Iwadate Y. Yumura S. (2008). Actin-based Propulsive Forces and Myosin-II-Based Contractile Forces in migrating Dictyostelium Cells. J. Cell. Sci. 121, 1314–1324. 10.1242/jcs.021576 18388319

[B53] Jay P. Y. Pham P. A. Wong S. A. Elson E. L. (1995). A Mechanical Function of Myosin II in Cell Motility. J. Cell. Sci. 108, 387–393. 10.1242/JCS.108.1.387 7738114

[B54] Jeon J. Kim D. Jeon T. J. (2021). Opposite Functions of RapA and RapC in Cell Adhesion and Migration in Dictyostelium. Animal Cells Syst. 25, 203–210. 10.1080/19768354.2021.1947372 PMC8370755 34413965

[B55] Jeon T. J. Lee D.-J. Lee S. Weeks G. Firtel R. A. (2007a). Regulation of Rap1 Activity by RapGAP1 Controls Cell Adhesion at the Front of Chemotaxing Cells. J. Cell. Biol. 179, 833–843. 10.1083/jcb.200705068 18039932 PMC2099181

[B56] Jeon T. J. Lee D.-J. Merlot S. Weeks G. Firtel R. A. (2007b). Rap1 Controls Cell Adhesion and Cell Motility through the Regulation of Myosin II. J. Cell. Biol. 176, 1021–1033. 10.1083/jcb.200607072 17371831 PMC2064086

[B57] Kamprad N. Witt H. Schröder M. Kreis C. T. Bäumchen O. Janshoff A. (2018). Adhesion Strategies of Dictyostelium discoideum- a Force Spectroscopy Study. Nanoscale 10, 22504–22519. 10.1039/c8nr07107a 30480299

[B58] Kang R. Kae H. Ip H. Spiegelman G. B. Weeks G. (2002). Evidence for a Role for the Dictyostelium Rap1 in Cell Viability and the Response to Osmotic Stress. J. Cell. Sci. 115, 3675–3682. 10.1242/jcs.00039 12186953

[B59] Kang S. Tice A. K. Stairs C. W. Jones R. E. Lahr D. J. G. Brown M. W. (2021). The Integrin-Mediated Adhesive Complex in the Ancestor of Animals, Fungi, and Amoebae. Curr. Biol. 31, 3073–3085. e3. 10.1016/J.CUB.2021.04.076 34077702

[B60] Kelsey J. S. Blumberg D. D. (2013). A SAP Domain-Containing Protein Shuttles between the Nucleus and Cell Membranes and Plays a Role in Adhesion and Migration in D. discoideum. Biol. Open 2, 396–406. 10.1242/bio.20133889 23616924 PMC3625868

[B61] Kim D. Kim W. Jeon T. J. (2021). Reversible Function of RapA with the C-Terminus of RapC in Dictyostelium. J. Microbiol. 59, 848–853. 10.1007/s12275-021-1400-5 34449058

[B62] Kim H. Lee M.-R. Jeon T. J. (2017). Loss of FrmB Results in Increased Size of Developmental Structures during the Multicellular Development of Dictyostelium Cells. J. Microbiol. 55, 730–736. 10.1007/s12275-017-7221-x 28865076

[B63] Kolanus W. Nagel W. Schiller B. Zeitlmann L. Godar S. Stockinger H. (1996). αLβ2 Integrin/LFA-1 Binding to ICAM-1 Induced by Cytohesin-1, a Cytoplasmic Regulatory Molecule. Cell. 86, 233–242. 10.1016/S0092-8674(00)80095-1 8706128

[B64] Kortholt A. Bolourani P. Rehmann H. Keizer-Gunnink I. Weeks G. Wittinghofer A. (2010). A Rap/Phosphatidylinositol 3-Kinase Pathway Controls Pseudopod Formation. Mol. Biol. Cell 21, 936–945. 10.1091/mbc.e09-03-0177 20089846 PMC2836974

[B65] Kortholt A. Rehmann H. Kae H. Bosgraaf L. Keizer-Gunnink I. Weeks G. (2006). Characterization of the GbpD-Activated Rap1 Pathway Regulating Adhesion and Cell Polarity in Dictyostelium discoideum. J. Biol. Chem. 281, 23367–23376. 10.1074/jbc.M600804200 16769729

[B66] Kowal A. S. Chisholm R. L. (2011). Uncovering a Role for the Tail of the Dictyostelium discoideum Sada Protein in Cell-Substrate Adhesion. Eukaryot. Cell. 10, 662–671. 10.1128/EC.00221-10 21441344 PMC3127660

[B67] Kreitmeier M. Gerisch G. Heizer C. Müller-Taubenberger A. (1995). A Talin Homologue of Dictyostelium Rapidly Assembles at the Leading Edge of Cells in Response to Chemoattractant. J. Cell. Biol. 129, 179–188. 10.1083/jcb.129.1.179 7698984 PMC2120370

[B68] Lagarrigue F. Kim C. Ginsberg M. H. (2016). The Rap1-RIAM-Talin axis of Integrin Activation and Blood Cell Function. Blood 128, 479–487. 10.1182/blood-2015-12-638700 27207789 PMC4965904

[B69] Lampert T. J. Kamprad N. Edwards M. Borleis J. Watson A. J. Tarantola M. (2017). Shear Force-Based Genetic Screen Reveals Negative Regulators of Cell Adhesion and Protrusive Activity. Proc. Natl. Acad. Sci. U.S.A. 114, E7727–E7736. 10.1073/pnas.1616600114 28847951 PMC5603988

[B70] Legerstee K. Houtsmuller A. (2021). A Layered View on Focal Adhesions. Biology 10, 1189. 10.3390/biology10111189 34827182 PMC8614905

[B71] Linkner J. Witte G. Zhao H. Junemann A. Nordholz B. Runge-Wollmann P. (2014). The Inverse BAR-Domain Protein IBARa Drives Membrane Remodelling to Control Osmoregulation, Phagocytosis and Cytokinesis. J. Cell. Sci. 127, 1279–1292. 10.1242/jcs.140756 24463811

[B72] Liu C.-I. Cheng T.-L. Chen S.-Z. Huang Y.-C. Chang W.-T. (2005). LrrA, a Novel Leucine-Rich Repeat Protein Involved in Cytoskeleton Remodeling, Is Required for Multicellular Morphogenesis in Dictyostelium discoideum. Dev. Biol. 285, 238–251. 10.1016/j.ydbio.2005.05.045 16051212

[B73] Liu Y.-J. Le Berre M. Lautenschlaeger F. Maiuri P. Callan-Jones A. Heuzé M. (2015). Confinement and Low Adhesion Induce Fast Amoeboid Migration of Slow Mesenchymal Cells. Cell 160, 659–672. 10.1016/j.cell.2015.01.007 25679760

[B74] Liu Y. Makarova K. S. Huang W.-C. Wolf Y. I. Nikolskaya A. N. Zhang X. (2021). Expanded Diversity of Asgard Archaea and Their Relationships with Eukaryotes. Nature 593, 553–557. 10.1038/s41586-021-03494-3 33911286 PMC11165668

[B75] Loomis W. F. Fuller D. Gutierrez E. Groisman A. Rappel W.-J. (2012). Innate Non-specific Cell Substratum Adhesion. PLoS One 7, e42033. 10.1371/journal.pone.0042033 22952588 PMC3432024

[B76] McLeod S. J. Shum A. J. Lee R. L. Takei F. Gold M. R. (2004). The Rap GTPases Regulate Integrin-Mediated Adhesion, Cell Spreading, Actin Polymerization, and Pyk2 Tyrosine Phosphorylation in B Lymphocytes. J. Biol. Chem. 279, 12009–12019. 10.1074/jbc.M313098200 14701796

[B77] Müller-Taubenberger A. Ishikawa-Ankerhold H. C. Kastner P. M. Burghardt E. Gerisch G. (2009). The STE Group Kinase SepA Controls Cleavage Furrow Formation in Dictyostelium. Cell Motil. Cytoskelet. 66, 929–939. 10.1002/cm.20386 19479821

[B78] Murray P. S. Zaidel-Bar R. (2014). Pre-metazoan Origins and Evolution of the Cadherin Adhesome. Biol. Open 3, 1183–1195. 10.1242/BIO.20149761 25395670 PMC4265756

[B79] Nagasaki A. Kanada M. Uyeda T. Q. (2009). Cell Adhesion Molecules Regulate Contractile Ring-independent Cytokinesis in Dictyostelium discoideum. Cell Res. 19, 236–246. 10.1038/cr.2008.318 19065153

[B80] Niewöhner J. Weber I. Maniak M. Müller-Taubenberger A. Gerisch G. (1997). Talin-Null Cells of Dictyostelium Are Strongly Defective in Adhesion to Particle and Substrate Surfaces and Slightly Impaired in Cytokinesis. J. Cell Biol. 138, 349–361. 9230077 10.1083/jcb.138.2.349 PMC2138202

[B81] Owens N. F. Gingell D. Rutter P. R. (1987). Inhibition of Cell Adhesion by a Synthetic Polymer Adsorbed to Glass Shown under Defined Hydrodynamic Stress. J. Cell Sci. 87, 667–675. 10.1242/jcs.87.5.667 3312253

[B82] Owens N. F. Gingell D. Trommler A. (1988). Cell Adhesion to Hydroxyl Groups of a Monolayer Film. J. Cell Sci. 91 (Pt 2), 269–279. 10.1242/jcs.91.2.269 3267699

[B83] Park B. Shin D. Y. Jeon T. J. (2018b). CBP7 Interferes with the Multicellular Development of Dictyostelium Cells by Inhibiting Chemoattractant-Mediated Cell Aggregation. Mol. Cells 41, 103–109. 10.14348/molcells.2018.2170 29385672 PMC5824019

[B84] Park B. Kim H. Jeon T. J. (2018a). Loss of RapC Causes Defects in Cytokinesis, Cell Migration, and Multicellular Development of Dictyostelium. Biochem. Biophysical Res. Commun. 499, 783–789. 10.1016/j.bbrc.2018.03.223 29614268

[B85] Parkinson K. Bolourani P. Traynor D. Aldren N. L. Kay R. R. Weeks G. (2009). Regulation of Rap1 Activity Is Required for Differential Adhesion, Cell-type Patterning and Morphogenesis in Dictyostelium. J. Cell. Sci. 122, 335–344. 10.1242/jcs.036822 19126673 PMC2724730

[B86] Parra-Acero H. Harcet M. Sánchez-Pons N. Casacuberta E. Brown N. H. Dudin O. (2020). Integrin-Mediated Adhesion in the Unicellular Holozoan Capsaspora owczarzaki. Curr. Biol. 30, 4270–4275. 10.1016/j.cub.2020.08.015 32857975

[B87] Patel H. König I. Tsujioka M. Frame M. C. Anderson K. I. Brunton V. G. (2008). The Multi-FERM-Domain-Containing Protein FrmA Is Required for Turnover of Paxillin-Adhesion Sites during Cell Migration of Dictyostelium. J. Cell Sci. 121, 1159–1164. 10.1242/jcs.021725 18349074

[B88] Patla I. Volberg T. Elad N. Hirschfeld-Warneken V. Grashoff C. Fässler R. (2010). Dissecting the Molecular Architecture of Integrin Adhesion Sites by Cryo-Electron Tomography. Nat. Cell. Biol. 12 (12), 909–915. 10.1038/ncb2095 20694000

[B89] Petersen K. J. Goodson H. V. Arthur A. L. Luxton G. W. G. Houdusse A. Titus M. A. (2016). MyTH4-FERM Myosins Have an Ancient and Conserved Role in Filopod Formation. Proc. Natl. Acad. Sci. U.S.A. 113, E8059–E8068. 10.1073/pnas.1615392113 27911821 PMC5167205

[B90] Plak K. Pots H. Van Haastert P. J. M. Kortholt A. (2016). Direct Interaction between TalinB and Rap1 Is Necessary for Adhesion of Dictyostelium Cells. BMC Cell Biol. 17, 1–8. 10.1186/s12860-015-0078-0 26744136 PMC4861126

[B91] Pribic J. Garcia R. Kong M. Brazill D. (2011). Paxillin and Phospholipase D Interact to Regulate Actin-Based Processes in dictyostelium Discoideum. Eukaryot. Cell 10, 977–984. 10.1128/EC.00282-10 21531871 PMC3147424

[B92] Rai A. Nöthe H. Tzvetkov N. Korenbaum E. Manstein D. J. (2011). Dictyostelium Dynamin B Modulates Cytoskeletal Structures and Membranous Organelles. Cell Mol. Life Sci. 68, 2751–2767. 10.1007/s00018-010-0590-5 21086149 PMC3142549

[B93] Rebstein P. J. Weeks G. Spiegelman G. B. (1993). Altered Morphology of Vegetative Amoebae Induced by Increased Expression of the Dictyostelium discoideum Ras-Related Gene rap 1. Dev. Genet. 14, 347–355. 10.1002/dvg.1020140504 7507418

[B94] Riley D. R. J. Khalil J. S. Pieters J. Naseem K. M. Rivero F. (2020). Coronin 1 Is Required for Integrin β2 Translocation in Platelets. Int. J. Mol. Sci. 21, 356–420. 10.3390/ijms21010356 PMC6982036 31948107

[B95] Schirenbeck A. Bretschneider T. Arasada R. Schleicher M. Faix J. (2005). The Diaphanous-Related Formin dDia2 Is Required for the Formation and Maintenance of Filopodia. Nat. Cell Biol. 7, 619–625. 10.1038/ncb1266 15908944

[B96] Sebé-Pedrós A. Roger A. J. Lang F. B. King N. Ruiz-Trillo I. (2010). Ancient Origin of the Integrin-Mediated Adhesion and Signaling Machinery. Proc. Natl. Acad. Sci. U.S.A. 107, 10142–10147. 10.1073/pnas.1002257107 20479219 PMC2890464

[B97] Shina M. C. Müller R. Blau-Wasser R. Glöckner G. Schleicher M. Eichinger L. (2010a). A Cytohesin Homolog in Dictyostelium Amoebae. PLoS One 5, e9378. 10.1371/journal.pone.0009378 20186335 PMC2826412

[B98] Shina M. C. Ünal C. Eichinger L. Müller-Taubenberger A. Schleicher M. Steinert M. (2010b). A Coronin7 Homolog with Functions in Actin-Driven Processes. J. Biol. Chem. 285, 9249–9261. 10.1074/jbc.M109.083725 20071332 PMC2838343

[B99] Simson R. Wallraff E. Faix J. Niewöhner J. Gerisch G. Sackmann E. (1998). Membrane Bending Modulus and Adhesion Energy of Wild-type and Mutant Cells of Dictyostelium Lacking Talin or Cortexillins. Biophysical J. 74, 514–522. 10.1016/S0006-3495(98)77808-7 PMC1299403 9449351

[B100] Tamura M. Gu J. Matsumoto K. Aota S.-i. Parsons R. Yamada K. M. (1998). Inhibition of Cell Migration, Spreading, and Focal Adhesions by Tumor Suppressor PTEN. Science 280, 1614–1617. 10.1126/science.280.5369.1614 9616126

[B101] Tarantola M. Bae A. Fuller D. Bodenschatz E. Rappel W.-J. Loomis W. F. (2014). Cell Substratum Adhesion during Early Development of Dictyostelium discoideum. PLoS One 9, e106574–7. 10.1371/journal.pone.0106574 25247557 PMC4172474

[B102] Titus M. A. (1999). A Class VII Unconventional Myosin Is Required for Phagocytosis. Curr. Biol. 9, 1297–1303. 10.1016/S0960-9822(00)80051-2 10574761

[B103] Todd I. Mellor J. S. Gingell D. (1988). Mapping Cell-Glass Contacts of Dictyostelium Amoebae by Total Internal Reflection Aqueous Fluorescence Overcomes a Basic Ambiguity of Interference Reflection Microscopy. J. Cell Sci. 89, 107–114. 10.1242/jcs.89.1.107 3417790

[B104] Tsujioka M. Yoshida K. Inouye K. (2004). Talin B Is Required for Force Transmission in Morphogenesis of Dictyostelium. EMBO J. 23, 2216–2225. 10.1038/sj.emboj.7600238 15141168 PMC419915

[B105] Tsujioka M. Yoshida K. Nagasaki A. Yonemura S. Müller Taubenberger A. Uyeda T. Q. P. (2008). Overlapping Functions of the Two Talin Homologues in Dictyostelium. Eukaryot. Cell 7, 906–916. 10.1128/EC.00464-07 18375618 PMC2394970

[B106] Tsujioka M. Yumura S. Inouye K. Patel H. Ueda M. Yonemura S. (2012). Talin Couples the Actomyosin Cortex to the Plasma Membrane during Rear Retraction and Cytokinesis. Proc. Natl. Acad. Sci. U.S.A. 109, 12992–12997. 10.1073/pnas.1208296109 22826231 PMC3420178

[B107] Tuxworth R. I. Stephens S. Ryan Z. C. Titus M. A. (2005). Identification of a Myosin VII-Talin Complex. J. Biol. Chem. 280, 26557–26564. 10.1074/jbc.M503699200 15826949

[B108] Tuxworth R. I. Weber I. Wessels D. Addicks G. C. Soll D. R. Gerisch G. (2001). A Role for Myosin VII in Dynamic Cell Adhesion. Curr. Biol. 11, 318–329. 10.1016/S0960-9822(01)00097-5 11267868

[B109] Uchida K. S. K. Yumura S. (2004). Dynamics of Novel Feet of Dictyostelium Cells during Migration. J. Cell Sci. 117, 1443–1455. 10.1242/jcs.01015 15020673

[B110] Veltman D. M. King J. S. Machesky L. M. Insall R. H. (2012). SCAR Knockouts in Dictyostelium: WASP Assumes SCAR's Position and Upstream Regulators in Pseudopods. J. Cell Biol. 198, 501–508. 10.1083/jcb.201205058 22891261 PMC3514037

[B111] Weber I. Wallraff E. Albrecht R. Gerisch G. (1995). Motility and Substratum Adhesion of Dictyostelium Wild-type and Cytoskeletal Mutant Cells: A Study by RICM/bright-field Double-View Image Analysis. J. Cell Sci. 108, 1519–1530. 10.1242/jcs.108.4.1519 7615672

[B112] Wessels D. Vawter-Hugart H. Murray J. Soll D. R. (1994). Three-dimensional Dynamics of Pseudopod Formation and the Regulation of Turning during the Motility Cycle of Dictyostelium. Cell Motil. Cytoskelet. 27, 1–12. 10.1002/CM.970270102 8194106

[B113] Yumura S. Kitanishi-Yumura T. (1990). Fluorescence-mediated Visualization of Actin and Myosin Filaments in the Contractile Membrane-Cytoskeleton Complex of Dictyostelium discoideum. Cell Struct. Funct. 15, 355–364. 10.1247/csf.15.355 2085848

[B114] Zaidel-Bar R. (2009). Evolution of Complexity in the Integrin Adhesome. J. Cell Biol. 186, 317–321. 10.1083/JCB.200811067 19667126 PMC2728394

[B115] Zhang H. Chang Y.-C. Brennan M. L. Wu J. (2014). The Structure of RAP1 in Complex with RIAM Reveals Specificity Determinants and Recruitment Mechanism. Jpn. J. Clin. Oncol. 6, 128–139. 10.1093/jmcb/mjt044 PMC4817127 24287201

[B116] Zhu L. Yang J. Bromberger T. Holly A. Lu F. Liu H. (2017). Structure of Rap1b Bound to Talin Reveals a Pathway for Triggering Integrin Activation. Nat. Commun. 8, 1–11. 10.1038/s41467-017-01822-8 29170462 PMC5701058

[B117] Zidovska A. Sackmann E. (2006). Brownian Motion of Nucleated Cell Envelopes Impedes Adhesion. Phys. Rev. Lett. 96, 5–8. 10.1103/PhysRevLett.96.048103 16486899

